# Identity development and the transition to adulthood under polycrisis: a one-year longitudinal study of emerging adults

**DOI:** 10.3389/fpubh.2026.1958411

**Published:** 2026-10-14

**Authors:** Anna Wendołowska, Katarzyna Sitnik-Warchulska, Weronika Kałwak, Aleksandra Gruszka, Dorota Czyżowska, Bernadetta Izydorczyk

**Affiliations:** 1 Faculty of Philosophy, Institute of Psychology, Jagiellonian University, Kraków, Poland; 2 Faculty of Management and Social Communication, Institute of Applied Psychology, Jagiellonian University, Kraków, Poland

**Keywords:** emerging adulthood, identity development, mental health, polycrisis, resilience

## Abstract

**Background:**

Contemporary emerging adults are navigating the transition to adulthood under conditions of prolonged societal uncertainty associated with multiple concurrent global crises. Although psychological stress has been extensively linked to poorer mental health, considerably less is known about its prospective associations with identity development and adulthood attainment.

**Objective:**

This longitudinal study examined whether baseline psychological stress burden prospectively predicted identity development, adulthood attainment, and resilience-related resources over 1 year, and whether baseline resilience-related resources prospectively predicted identity and adulthood attainment independently of stress burden.

**Methods:**

Data were drawn from a nationwide longitudinal panel of 831 Polish emerging adults (18–29 years) assessed at two time points separated by 1 year. Psychological stress burden was modelled as a latent factor indicated by perceived stress, adjustment-disorder symptoms, and polycrisis-related adjustment difficulties. Identity development was assessed using the five-dimensional model of identity formation, alongside measures of resilience-related resources and subjective and objective adulthood. Longitudinal hypotheses were examined using autoregressive structural equation models controlling for baseline levels of the respective outcomes and relevant sociodemographic covariates. Subjective adulthood was modelled as a binary outcome using probit structural equation modelling.

**Results:**

All developmental outcomes showed significant rank-order stability across the follow-up period, while mean-level changes were generally small. Higher baseline psychological stress burden prospectively predicted greater ruminative exploration, lower commitment making, and lower identification with commitment, but was not significantly associated with exploration in breadth, exploration in depth, resilience-related resources, objective adulthood, or subjective adulthood. Higher baseline resilience-related resources prospectively predicted greater exploration in breadth, commitment making, exploration in depth, and identification with commitment, independently of baseline psychological stress burden, but did not significantly predict ruminative exploration, objective or subjective adulthood.

**Conclusion:**

Psychological stress burden and resilience-related resources showed distinct prospective associations with identity development, whereas neither significantly predicted objective or subjective adulthood after accounting for baseline levels and covariates. Higher psychological stress burden was associated with greater ruminative exploration and weaker commitment-related processes, whereas resilience-related resources were associated with both adaptive exploration and commitment processes. These findings suggest that, over the one-year interval examined, prospective associations with psychological stress burden and resilience-related resources were concentrated primarily within identity development rather than broader adulthood attainment. Longer multiwave studies are needed to determine how these associations unfold across the transition to adulthood.

## 1 Introduction

### 1.1 Emerging adulthood and identity development

Emerging adulthood, typically spanning the ages of 18 to 29 years, represents a distinct developmental period situated between adolescence and adulthood, characterized by profound psychological, cognitive, and social changes (1, 2). Unlike previous developmental stages, emerging adulthood is not defined primarily by chronological age but by active engagement in developmental tasks that gradually establish adult identity, autonomy, and social roles. During this period, young people complete their education, enter the labour market, establish financial independence, leave the parental home, develop long-term romantic relationships, and begin to assume enduring social responsibilities. These transitions create unique opportunities for personal growth, self-realization, and autonomy, but simultaneously expose individuals to numerous developmental challenges requiring substantial psychological adaptation (1–3).

Arnett (1, 2) proposed that emerging adulthood is distinguished by five defining characteristics: identity exploration, instability, self-focus, feeling “in-between,” and optimism regarding future possibilities. Identity exploration is particularly central, as emerging adults actively evaluate educational, occupational, relational, and ideological alternatives while gradually establishing personally meaningful commitments. At the same time, instability resulting from frequent changes in education, employment, residence, and interpersonal relationships often creates considerable uncertainty. Many emerging adults report feeling neither adolescents nor fully adult, reflecting the developmental experience of being “in-between” established life stages (1, 2). Although these characteristics provide unique opportunities for exploration and personal development, they also make emerging adulthood a period of heightened vulnerability to stress and psychological difficulties (4).

The developmental demands associated with this life stage require young people to make numerous consequential decisions while possessing relatively limited experience in managing adult roles and responsibilities. Consequently, emerging adulthood has been associated with elevated psychological distress, fluctuations in emotional wellbeing, and an increased incidence of mental health problems compared with many other stages of the life course (5–7). Nevertheless, developmental theory emphasizes that uncertainty and instability are not inherently maladaptive but constitute normative features of the transition to adulthood that facilitate exploration, learning, and identity formation (1, 2). An important question, therefore, is how contemporary conditions of prolonged societal uncertainty are associated with the developmental processes through which young people negotiate this transition.

Identity development represents a central developmental task during emerging adulthood. Building on Erikson’s (8) psychosocial theory, contemporary identity models conceptualize identity formation as a dynamic and lifelong process involving continuous interactions between exploration and commitment rather than a single developmental achievement (9–11). Exploration enables individuals to evaluate alternative life directions before making important commitments, whereas commitment reflects the establishment and internalization of personally meaningful choices. However, identity exploration may also assume a maladaptive form characterized by persistent indecision, repetitive self-doubt, and ruminative exploration that hinders the formation of stable commitments (9, 12). Recent systematic reviews demonstrate that adaptive identity development has been consistently associated with psychological wellbeing during adolescence and emerging adulthood, whereas identity confusion and prolonged maladaptive exploration are consistently associated with depression, anxiety, loneliness, and poorer psychosocial functioning (13–15). These findings suggest that identity development represents not only a normative developmental task but also an important domain relevant to public mental health.

Identity development is closely intertwined with the broader transition to adulthood. Traditionally, adulthood was defined primarily by objective role transitions, including completing education, obtaining stable employment, achieving financial independence, leaving the parental home, establishing long-term relationships, and becoming a parent. Contemporary developmental perspectives, however, emphasize that adulthood also involves subjective perceptions of autonomy, responsibility, and self-sufficiency (1). Consequently, objective and subjective adulthood represent complementary but distinct dimensions of developmental adaptation. As educational pathways, labour-market participation, and family formation have become increasingly diverse and delayed, objective markers of adulthood no longer necessarily coincide with individuals’ subjective perceptions of having become an adult (16). Examining both objective and subjective indicators therefore provides a more comprehensive understanding of developmental adaptation during emerging adulthood than considering either dimension in isolation.

### 1.2 Development under polycrisis and psychological stress burden

Although uncertainty and instability are normative features of emerging adulthood, contemporary cohorts are negotiating this developmental transition under social conditions that differ fundamentally from those experienced by previous generations. Rather than confronting isolated developmental challenges, today’s emerging adults are simultaneously exposed to multiple large-scale societal crises, including the COVID-19 pandemic, economic instability, inflation, geopolitical conflicts, climate change, and rapid technological transformation. Increasingly, these interacting challenges have been conceptualized as a polycrisis, reflecting the synergistic interaction of multiple crises whose combined effects exceed the sum of their individual impacts (17).

Poland provides a particularly informative Central and Eastern European context for examining development under polycrisis. Young people entering adulthood experienced the consequences of the COVID-19 pandemic alongside the war in neighbouring Ukraine and the associated refugee and geopolitical crisis, while also facing economic uncertainty characterized by inflation and instability in housing and labour markets (18). Research among Polish young adults indicates that these crises were experienced as overlapping rather than isolated sources of uncertainty, with war- and economic-crisis stress being particularly salient and unfavourable financial circumstances associated with greater crisis-related stress (19). The Polish setting therefore represents a context in which several macrosystemic sources of uncertainty converged during the transition to adulthood, while remaining a specific Central and Eastern European case rather than a proxy for the experiences of emerging adults internationally.

The concept of polycrisis provides a useful framework for considering how emerging adults navigate development in a social context characterized by multiple, overlapping sources of uncertainty. Rather than representing temporary disruptions, interconnected societal crises may contribute to persistent uncertainty regarding education, employment, housing, financial security, interpersonal relationships, and future life opportunities. Wood et al. (20) argued that emerging adulthood has traditionally been understood as the “volitional years,” during which young people possess sufficient opportunities to explore identities and experiment with different life pathways before making enduring commitments. However, increasing economic insecurity, social inequalities, and prolonged societal instability may substantially constrain these developmental opportunities and shape the context within which identity development and the transition to adulthood occur. Similarly, Côté (21) argued that contemporary structural conditions increasingly shape developmental opportunities, reducing young people’s capacity to navigate the transition to adulthood solely through individual agency.

Recent conceptual work has increasingly emphasized prolonged societal uncertainty as an important developmental context for contemporary young people. The *Lancet Psychiatry Commission on Youth Mental Health* highlights that contemporary young people are negotiating developmental transitions amid unprecedented social, economic, and environmental transformations that have fundamentally reshaped the experience of becoming an adult (22). Likewise, recent work by Pinheiro et al. (23) emphasizes that cumulative societal crises should be viewed not simply as external stressors but as developmental contexts influencing identity formation, social integration, and mental health across the life course.

A growing body of empirical evidence demonstrates that contemporary societal crises have been accompanied by increased levels of stress, anxiety, depression, loneliness, and adjustment difficulties among young adults. However, as highlighted by recent systematic reviews, the overwhelming majority of studies have focused on mental health outcomes, with relatively little attention devoted to developmental processes themselves (14, 15). Consequently, although it is well established that emerging adults experience elevated psychological distress during periods of societal uncertainty, considerably less is known about whether psychological stress is prospectively associated with identity development and the transition to adulthood over time. Distinguishing psychological adjustment from developmental adaptation is particularly important because temporary emotional distress does not necessarily imply disruption of the developmental processes through which individuals establish enduring identities, assume adult roles, and build the foundations for later adult functioning.

From a developmental perspective, psychological stress burden may be associated with how emerging adults negotiate age-salient developmental tasks. Contemporary stress theories propose that chronic and unpredictable stress places demands on cognitive, emotional, and behavioural resources involved in adaptive functioning, thereby increasing vulnerability to maladaptive developmental outcomes (24, 25). Emerging adulthood may be particularly sensitive to these influences because many developmental tasks characteristic of this period—including establishing educational and occupational pathways, achieving financial independence, forming stable intimate relationships, and consolidating personal identity—require sustained self-regulation, future-oriented planning, and effective emotion regulation (2, 10). Higher psychological stress burden may therefore be associated with less adaptive identity processes and slower progress toward adulthood, although these prospective associations remain insufficiently understood.

From an ecological-developmental perspective, however, macro-level societal instability should not be assumed to translate directly into individual psychological or developmental outcomes. Rogers et al. (26) emphasize that macrosystemic forces are not merely distal background conditions but shape development through institutions, opportunity structures, cultural meanings, and the proximal contexts in which everyday development occurs. Similarly, Collins (27) proposes that instability in social, economic, and political structures may generate psychological insecurity by reducing the predictability of future conditions and constraining individuals’ capacity for planning and goal-directed action. Accordingly, polycrisis is conceptualized here as a macrosystemic developmental context, whereas its individual consequences depend on exposure, appraisal, stress-related adjustment, and available resources. In the present study, psychological stress burden refers to the shared variance across perceived stress, adjustment-disorder symptoms, and polycrisis-related adjustment difficulties. It is therefore conceptualized as an individual-level psychological burden occurring within a broader polycrisis context, rather than as a direct measure of exposure to macro-level crises.

### 1.3 Resilience-related resources

At the same time, increasing evidence suggests that not all emerging adults respond similarly to prolonged adversity. Resilience is now widely conceptualized as a dynamic developmental process involving successful adaptation despite significant adversity rather than as a stable personality trait or simple resistance to stress (28, 29). Contemporary developmental theories emphasize that resilience emerges through interactions between individual capacities and environmental resources, enabling individuals to maintain adaptive functioning despite substantial challenges (30). From this perspective, resilience reflects the capacity to preserve developmental functioning under adverse circumstances rather than the absence of psychological distress.

The developmental role of resilience can also be understood within the framework of Conservation of Resources Theory (31), which proposes that individuals strive to acquire, preserve, and restore psychological, social, and material resources necessary for successful adaptation. Chronic stress may threaten these resources, whereas resource preservation and replenishment may support adaptive functioning under conditions of prolonged adversity. During emerging adulthood, such resources may include optimism, self-efficacy, emotion regulation, supportive interpersonal relationships, and future orientation—all of which facilitate successful navigation of educational, occupational, and relational transitions. Accordingly, resilience-related resources may not eliminate exposure to stress but may support young adults in continuing to progress toward developmental goals despite persistent uncertainty. Within this broader process-oriented conceptualization, resilience-related personal, family, and social resources may contribute to successful adaptation under adversity. The present study does not directly assess resilience as a dynamic process. Instead, it assesses personal, family, and social resources associated with resilient functioning. Accordingly, we use the term *resilience-related resources* when referring to the construct measured in the present study.

Recent evidence supports this developmental perspective. Reviews indicate that resilience represents one of the strongest protective factors associated with psychological wellbeing, life satisfaction, and successful psychosocial adaptation among emerging adults (32–34). Similarly, positive psychology frameworks emphasize that strengths such as optimism, purpose in life, adaptive coping, and positive relationships contribute to flourishing during the transition to adulthood (35, 36). Beyond its established associations with psychological wellbeing, resilience-related resources may also be prospectively associated with developmental functioning by supporting adaptive self-regulation, sustained goal pursuit, and successful integration of developmental experiences. In this sense, resilience-related resources may be associated with emerging adults’ capacity to maintain identity commitments and pursue longer-term developmental goals, and successfully negotiate the transition to adulthood despite prolonged societal uncertainty.

Despite the rapidly expanding literature on stress, resilience, and mental health, important gaps remain. Most studies have examined psychological symptoms rather than developmental outcomes, have relied on cross-sectional designs, or have investigated individual stressors in isolation. Consequently, relatively little is known about whether psychological stress burden is prospectively associated with identity development and adulthood attainment after accounting for baseline levels of these outcomes. Likewise, although resilience-related resources have repeatedly been associated with positive psychological functioning, considerably fewer longitudinal studies have examined their prospective associations with identity development and adulthood attainment while accounting for psychological stress burden. Addressing these questions may help distinguish factors associated with psychological adjustment from those associated with developmental processes during emerging adulthood.

### 1.4 Study aim and hypotheses

The present longitudinal study examined whether psychological stress burden and resilience-related resources were prospectively associated with developmental outcomes over a one-year period. Specifically, we investigated whether psychological stress burden, modelled as a latent construct reflected by perceived stress, adjustment disorder symptoms, and polycrisis-related adjustment difficulties, predicted follow-up identity development, resilience-related resources, and objective and subjective adulthood after accounting for baseline levels of the respective outcomes. We further examined whether baseline resilience-related resources predicted follow-up identity development and objective and subjective adulthood after accounting for baseline developmental outcomes and psychological stress burden. Accordingly, the following hypotheses were tested:

*H1*: Higher baseline psychological stress burden was expected to predict less adaptive identity development, lower objective adulthood attainment, a weaker subjective sense of adulthood, and lower resilience-related resources at follow-up, after controlling for baseline levels of the respective outcomes.

*H2*: Higher baseline resilience-related resources were expected to predict more adaptive identity development, greater objective adulthood attainment, and a stronger subjective sense of adulthood at follow-up, after controlling for baseline levels of the respective developmental outcomes and baseline psychological stress burden.

## 2 Methods

### 2.1 Procedure

Data were collected as part of a nationwide longitudinal study investigating identity development, transition to adulthood, and mental health among emerging adults in Poland. Participants were recruited through Pollster, a professional research panel provider, and completed the surveys online. Participants were compensated through the provider’s standard respondent-reward system and received PLN 5.80 (58 panel points) for completing each survey. Information about the available compensation and expected survey duration was provided in the invitation email and through participants’ individual panel accounts. Prior to data collection, the research team tested the survey and estimated a completion time of approximately 20–30 min. The median completion time was 28 min at T1 and 26 min at T2. The first wave (T1) was conducted in 2024. Participants were eligible if they were between 18 and 29 years of age, resided in Poland, and provided informed consent. At T1, 2,740 respondents completed the survey. Of these, 15 were excluded because they did not provide informed consent, 61 because they were outside the target age range, 259 because of insufficient questionnaire completion, and 10 because of straightlining, resulting in an analytic baseline sample of 2,395 participants. Approximately 1 year later, participants who had agreed to be re-contacted were invited to complete the follow-up survey (T2). A total of 1,004 respondents completed the follow-up survey. After matching responses across measurement occasions using a unique participant identifier, 6 respondents were excluded because they did not provide informed consent, 101 because of insufficient questionnaire completion, 5 because of straightlining, and 61 because they failed an attention-check item. The final longitudinal sample consisted of 831 participants, corresponding to 34.7% of the T1 analytic sample.

The study was approved by the Ethics Committee of the Institute of Psychology, Jagiellonian University (approval no. 221.0042.26.2023) and was conducted in accordance with the ethical principles of the Declaration of Helsinki. All participants provided informed consent electronically before completing the survey.

### 2.2 Participants

The final longitudinal sample comprised 831 emerging adults aged 18–29 years (*M* = 25.08, SD = 3.18). Using post-stratification weights, 49.6% were women. Overall, 35.6% were students, 67.3% were employed, and 53.2% were involved in a romantic relationship. Approximately 40.2% resided in rural areas, 23.8% in towns with fewer than 50,000 inhabitants, 13.1% in medium-sized cities, and 23.0% in large cities (>150,000 inhabitants). Finally, 61.6% reported that they considered themselves adults. Additional demographic characteristics are provided in Table 1. From the 2,395 eligible participants at baseline, 831 completed the follow-up survey and met all inclusion criteria. Attrition was selective with respect to age, gender, and educational attainment, whereas place of residence did not differ significantly between retained and non-retained participants. Differences in the principal baseline psychological variables were generally small in magnitude. To assess the potential influence of selective retention, inverse-probability-of-retention weighted sensitivity analyses were conducted.

**Table 1:** Sample characteristics.

Characteristic	n/M (SD)	%/Range
Age at T1, years	25.08 (3.18)	18–29
Age at T2, years	26.08 (3.18)	19–30
Gender (weighted)		
Women		49.6
Men		50.4
Place of residence (weighted)		
Rural area		40.2
Town < 50,000 inhabitants		23.8
Town 50,000–150,000 inhabitants		13.1
City > 150,000 inhabitants		23.0
Student status (weighted)		
Student		35.6
Non-student		64.4
Employment status (weighted)		
Employed		67.3
Unemployed		25.5
Inactive by choice		7.2
Relationship status (weighted)		
Single		46.5
Non-marital relationship		35.2
Married		18.0
Other		0.2
Subjective adulthood (weighted)		
Yes		61.6
In some respects yes, in others no		37.7
No		0.7

### 2.3 Measures

Perceived stress was assessed using the 10-item Perceived Stress Scale [PSS-10; Cohen et al. (37); Polish adaptation: Juczyński and Ogińska-Bulik (38)]. The measure assesses the extent to which situations experienced during the preceding month were perceived as unpredictable, uncontrollable, and overwhelming (e.g., “In the last month, how often have you been upset because of something that happened unexpectedly?”). Participants responded using a 5-point scale ranging from 0 (never) to 4 (very often). Four positively worded items—Items 4, 5, 7, and 8—were reverse-coded, after which the items were summed. Possible scores range from 0 to 40, with higher scores indicating greater perceived stress. Internal consistency was good at both baseline, *α* = 0.84, and follow-up, *α* = 0.83.

Adjustment disorder symptoms associated with individual life stressors were measured with the International Adjustment Disorder Questionnaire [IADQ; Shevlin et al. (39)]. The questionnaire follows the ICD-11 conceptualization of adjustment disorder and comprises a stressor checklist, six symptom items, and three functional-impairment items. Participants first indicated whether they had experienced potentially stressful life events, including financial difficulties, relationship problems, illness, and other significant stressors. The symptom section assesses the two core dimensions of adjustment disorder: preoccupation with the stressor (e.g., “I worry a lot more since the stressful event(s)”) and failure to adapt (e.g., “I find it difficult to relax and feel calm”), each represented by three items. Items are rated on a 5-point scale from 0 (not at all) to 4 (extremely), referring to the preceding month. The six symptom items were averaged, with higher scores indicating greater adjustment-disorder symptom severity. Internal consistency of the symptom score was excellent at baseline, *α* = 0.91, and follow-up, *α* = 0.91.

Adjustment difficulties specifically associated with concurrent global and socio-environmental crises were assessed using the International Adjustment Disorder Questionnaire–Polycrisis Version (IADQ-PC), an adaptation of the IADQ developed for the present research programme and psychometrically evaluated in a large Polish sample from the broader project from which the present longitudinal sample was drawn (40). The IADQ-PC preserves the ICD-11 symptom structure and diagnostic framework of the original IADQ while anchoring symptom assessment specifically to contemporary socio-environmental stressors. Participants first indicated current exposure to crisis-related stressors, including war or armed conflict, pandemics, climate change and environmental degradation, global economic instability, migration, and discrimination and social diversity, and identified the stressor(s) affecting them most strongly. Six symptom items then assessed the two core dimensions of adjustment disorder: Preoccupation (three items; e.g. “I worry a lot more since the stressful event(s).”) and Failure to Adapt (three items; e.g. “I find it difficult to relax and feel calm”). Symptoms were rated with reference to the most distressing crisis-related stressor(s) during the preceding month on a 5-point scale ranging from 0 (*not at all*) to 4 (*extremely*). A total symptom severity score was derived from the six symptom items, with higher scores indicating greater polycrisis-related adjustment difficulties. The questionnaire additionally assesses symptom onset within 1 month of the identified stressor(s) and functional impairment in social relationships, work or education, and other important areas of life, allowing classification of probable adjustment disorder according to the ICD-11-aligned diagnostic algorithm. A separate psychometric evaluation supported the theoretically expected two-factor structure of Preoccupation and Failure to Adapt, with standardized factor loadings ranging from 0.81 to 0.87 and 0.86 to 0.91, respectively (40). The total symptom score demonstrated excellent internal consistency (*α* = 0.91, *ω* = 0.95), and evidence was also obtained for measurement invariance across gender and for convergent, discriminant, criterion, and known-groups validity. In the present longitudinal sample, internal consistency was high at baseline (*α* = 0.89) and excellent at follow-up (*α* = 0.92).

Identity development was assessed using the Polish version of the Dimensions of Identity Development Scale [DIDS; Luyckx et al. (10); Polish adaptation: Cieciuch (41)]. The questionnaire contains 25 items measuring five identity processes: exploration in breadth, commitment making, exploration in depth, identification with commitment, and ruminative exploration. Each dimension consists of five items. Participants rated their agreement with each statement on a 5-point scale ranging from 1 (strongly disagree) to 5 (strongly agree). Separate mean scores were calculated for each dimension; no overall identity score was computed. Higher scores indicate a higher level of the respective identity process. Internal consistency was high for most dimensions at baseline and follow-up (from 0.77 to 0.95). All five identity dimensions were included as outcomes in the primary longitudinal analyses. Because no overall identity score was computed, each dimension was examined separately within the joint autoregressive SEM.

Resilience-related resources were assessed using the 26-item Resilience Assessment Questionnaire [KOP-26; Gąsior et al. (42)]. The instrument was developed for adults and conceptualizes resilience through personal, family, and social competencies. Participants indicated their agreement with each statement on a 5-point scale (e.g., “In difficult situations, I can always count on someone in my family.”). Item responses were averaged, with higher scores indicating greater resilience-related resources. The total score demonstrated excellent internal consistency at baseline, *α* = 0.94, and follow-up, *α* = 0.94. In the present study, KOP-26 scores were interpreted as indicators of resilience-related personal, family, and social resources rather than as a direct measure of resilience as a dynamic process of successful adaptation under adversity.

Objective adulthood attainment was assessed using an adapted version of the Achieved Criteria for Adulthood measure based on Nelson and Barry (43). The items represent several domains of the transition to adulthood, including independence, interdependence, role transitions, norm compliance, biological and chronological transitions, and family capacities. Examples include financial independence from parents, accepting responsibility for the consequences of one’s actions, establishing a long-term career, maintaining a committed relationship, and being capable of supporting and caring for a family. The adapted measure comprised 34 applicable criteria. For 23 items, participants indicated the extent to which each criterion currently applied to them using a three-category response scale (*very true* = 1, *somewhat true* = 2, *not true* = 3). The remaining 11 items used a yes/no response format (*yes, applies to me* = 1; *no, does not apply to me* = 2). All items were reverse-coded prior to analysis so that higher values consistently indicated greater criterion attainment. Thus, the three-category items were recoded as 3 = very true, 2 = rather true, and 1 = not true, whereas the binary items were recoded as 2 = yes, applies to me and 1 = no, does not apply to me. Items were then aggregated within the seven theoretically defined adulthood domains. The seven domain scores served as indicators of a latent objective-adulthood factor at T1 and T2, with higher levels of the latent factor representing greater adulthood attainment. This specification was supported by additional analyses indicating a predominantly unidimensional structure: a one-factor model showed acceptable fit (CFI = 0.968, TLI = 0.952, RMSEA = 0.060, SRMR = 0.032), and parallel analysis likewise supported a one-factor solution. Higher factor scores therefore reflected greater overall attainment of adulthood criteria across the seven domains. The measure demonstrated good internal consistency at baseline, *α* = 0.85, and follow-up, *α* = 0.85.

Subjective adulthood was assessed with a single item asking participants whether they considered themselves to have reached adulthood. Response options were (a) yes; (b) in some respects yes and in some respects no; (c) no. This approach follows prior research distinguishing individuals who perceive themselves as adults from those who regard themselves as still transitioning into adulthood. For the primary analyses, responses were dichotomized as 1 = yes and 0 = in some respects yes and in some respects no/no; responses of *in some respects yes and in some respects no* and *no* were combined because only five participants selected *no* at follow-up. The resulting binary variable distinguished participants who regarded themselves as fully adult from those who did not. Internal consistency is not applicable to a single-item measure. Given the theoretical distinction between feeling “in-between” and not feeling adult, the implications of this dichotomization are considered in the Limitations section.

Positive mental health was measured using the 14-item Mental Health Continuum–Short Form [MHC-SF; Keyes (44); Polish validation: Karaś et al. (45)]. The instrument assesses emotional, social, and psychological wellbeing. Participants indicated how often they had experienced each aspect of wellbeing using a 6-point frequency scale (e.g., “During the past month, how often did you feel happy”). Higher scores indicate better positive mental health. The total score demonstrated excellent internal consistency at baseline, *α* = 0.95, and follow-up, *α* = 0.96. Positive mental health was not a primary study outcome but was retained as a baseline predictor in the inverse-probability-of-retention model.

### 2.4 Analytic strategy

All preliminary and descriptive analyses were conducted using Stata 18 (StataCorp LLC, College Station, TX, USA). Descriptive statistics were calculated for all study variables, including means, standard deviations, observed ranges, and indices of internal consistency (Cronbach’s α and McDonald’s ω). Pearson correlation coefficients were computed to examine bivariate associations among the study variables, and gender differences were evaluated using Welch’s independent-samples *t*-tests. Effect sizes were expressed as Cohen’s *d* together with 95% confidence intervals. Mean-level T1–T2 changes were examined using paired-samples *t*-tests. Standardized mean change was calculated by dividing the mean T1–T2 difference by the baseline (T1) standard deviation. Post-stratification weights based on age × gender, educational attainment, and place of residence were used for descriptive statistics, whereas the primary longitudinal models were estimated in the unweighted longitudinal sample.

The primary longitudinal hypotheses were examined using autoregressive structural equation models (SEMs). Baseline psychological stress burden was represented as a latent factor indicated by perceived stress (PSS-10), adjustment-disorder symptoms (IADQ), and polycrisis-related adjustment difficulties (IADQ-PC). This factor was interpreted as an individual-level representation of psychological stress burden within the broader polycrisis context rather than as a measure of objective cumulative exposure to societal crises.

For identity development, all five T2 DIDS identity dimensions—exploration in breadth, commitment making, exploration in depth, identification with commitment, and ruminative exploration—were modelled jointly and regressed on their corresponding T1 levels and the focal baseline predictor. Covariances were specified among the identity dimensions within T1 and T2 and between the baseline latent psychological stress burden and the T1 identity dimensions. H1 models examined baseline psychological stress burden as the focal predictor, whereas H2 models examined baseline resilience-related resources while additionally accounting for baseline psychological stress burden.

Resilience-related resources were examined as an outcome under H1 in a separate autoregressive SEM, with T2 resilience-related resources regressed on their T1 level and baseline psychological stress burden. Objective adulthood was modelled as a latent construct indicated by the seven adulthood-criteria domains: independence, interdependence, role transitions, norm compliance, biological transitions, chronological transitions, and family capacities. A one-factor measurement model showed acceptable fit (CFI = 0.968, TLI = 0.952, RMSEA = 0.060, SRMR = 0.032), and parallel analysis likewise supported a one-factor representation. In the longitudinal SEMs, T2 latent adulthood attainment was regressed on its T1 level and the focal baseline predictor. Corresponding factor loadings were constrained to equality across T1 and T2, and residual covariances were specified between corresponding indicators across waves to account for indicator-specific stability over time.

Subjective adulthood was analysed separately because of its categorical nature. Because only five participants selected the “not adult” category at follow-up, responses were dichotomized as fully adult (1) versus in-between/not adult (0). Subjective adulthood was analysed using an autoregressive probit SEM with weighted least squares mean- and variance-adjusted (WLSMV) estimation. T2 subjective adulthood was regressed on T1 subjective adulthood and the focal baseline predictor. A complete measure-to-model mapping for the primary longitudinal analyses is provided in Supplementary Table S7.

All longitudinal models were adjusted for baseline age, gender, educational attainment, place of residence, student status, employment status, relationship status, and financial situation. Educational attainment was grouped into low (primary, lower-secondary, or vocational), medium (secondary or post-secondary), and higher (bachelor’s or master’s) education. Relationship status was categorized as single, in a non-marital relationship, or married; the small “other” category was combined with the single category. Financial situation was categorized as difficult (responses 1–3), good (responses 4–5), or very good (response 6).

Missing data were handled using multiple imputation by chained equations (MICE). SEMs for continuous outcomes were estimated in R using the *lavaan* package with robust maximum-likelihood estimation (MLR), whereas the categorical subjective-adulthood models were estimated using WLSMV. Standardized coefficients (β) with 95% confidence intervals are reported for all longitudinal models. For the binary subjective-adulthood outcome, *R*^2^ was computed on the underlying latent-response scale, representing the proportion of variance in the latent continuous response underlying the observed binary outcome explained by the predictors. Model fit was evaluated using the comparative fit index (CFI), Tucker–Lewis index (TLI), root mean square error of approximation (RMSEA), and standardized root mean square residual (SRMR).

To control the false discovery rate arising from multiple focal tests, the Benjamini–Hochberg procedure (62) was applied separately to the five focal DIDS coefficients within H1 and the five focal DIDS coefficients within H2. Resilience-related resources, objective-adulthood, and subjective-adulthood outcomes were not included in the BH-FDR correction. Autoregressive coefficients and covariate effects were likewise not included. Statistical significance was evaluated using two-tailed tests with an alpha level of 0.05.

Sensitivity analyses were conducted to evaluate the robustness of the primary findings. First, to address selective attrition, probabilities of retention were estimated using logistic regression based on baseline age, gender, educational attainment, place of residence, perceived stress, the five identity dimensions, resilience-related resources, and positive mental health. Variables with baseline missing data, including the IADQ indicators, were not included in the retention model. The inverse estimated probabilities of retention were then used as weights to re-estimate the primary models. Second, to examine whether associations involving the latent psychological stress-burden factor were attributable primarily to a particular indicator, the latent factor was replaced by PSS-10, IADQ, and IADQ-PC scores entered simultaneously as separate predictors. Finally, for models showing comparatively weaker global fit in the covariate-adjusted specification, additional models without covariates were estimated to assess whether the focal associations were sensitive to covariate adjustment.

## 3 Results

### 3.1 Descriptive statistics and correlations

Descriptive statistics, indices of internal consistency were calculated for all study variables. Bivariate correlations among the study variables are presented in Supplementary Table S1. All multi-item measures demonstrated satisfactory to excellent internal consistency (Cronbach’s *α* = 0.77–0.96). As expected, the three indicators of psychological stress burden: perceived stress, adjustment disorder symptoms, and polycrisis-related adjustment-disorder symptoms were positively intercorrelated. Higher levels of these indicators were generally associated with lower resilience-related resources, less adaptive identity processes, lower adulthood attainment, and poorer positive mental health. In contrast, resilience-related resources were positively associated with adaptive identity processes and adulthood indicators. Because the primary hypotheses concerned prospective associations rather than cross-sectional relationships, the complete correlation matrix is provided in the Supplementary Tables S1-S7.

### 3.2 Gender differences

Gender differences were generally small in magnitude (Supplementary Table S2), with absolute effect sizes ranging from 0.01 to 0.57. Although several comparisons reached statistical significance, most effects were negligible or small. More pronounced differences were observed for selected objective-adulthood domains, with the largest difference observed for biological-transition criteria. Given the generally modest magnitude of these differences, gender was included as a covariate in the primary longitudinal models.

### 3.3 Rank-order stability of developmental outcomes

All longitudinal outcomes demonstrated significant rank-order stability across the approximately one-year follow-up period (Supplementary Table S5). Autoregressive coefficients ranged from *β* = 0.41 to *β* = 0.49 for the five identity dimensions and were higher for resilience-related resources (*β* = 0.72), objective adulthood (*β* = 0.73), and subjective adulthood (*β* = 0.77), all ps < 0.001. Specifically, stability coefficients for exploration in breadth, commitment making, exploration in depth, identification with commitment, and ruminative exploration were *β* = 0.41, 0.49, 0.41, 0.42, and 0.43, respectively. These coefficients indicate stability in participants’ relative standing on the study variables across waves; they do not, however, indicate an absence of mean-level change. Mean-level changes are therefore examined separately below.

### 3.4 Mean-level change across the follow-up

Mean-level changes across the one-year interval were generally small (Supplementary Table S4). Ruminative exploration decreased slightly from T1 (*M* = 3.42, SD = 1.05) to T2 (*M* = 3.33, SD = 1.01), mean difference = −0.09, 95% CI [−0.15, −0.02], *p* = 0.012, d = −0.08. Commitment making and identification with commitment showed no significant mean-level change, with effect sizes close to zero (ds = 0.03 and −0.01, respectively). The remaining identity dimensions likewise showed little mean-level change: exploration in depth (*d* = −0.03, *p* = 0.389) and exploration in breadth (*d* = −0.06, *p* = 0.068). Resilience-related resources also remained virtually unchanged at the mean level (MT1 = 3.47, MT2 = 3.47, *p* = 0.800, *d* = −0.01).

Objective adulthood showed small mean-level increases in several domains. Significant increases were observed for independence (*d* = 0.12, *p* < 0.001), family capacities (*d* = 0.06, *p* = 0.027), biological transitions (*d* = 0.11, *p* < 0.001), chronological transitions (*d* = 0.11, *p* < 0.001), and role transitions (*d* = 0.09, *p* < 0.001). Interdependence (*d* = 0.05, *p* = 0.079) and norm compliance (*d* = 0.02, *p* = 0.597) did not change significantly. Thus, where mean-level changes in objective adulthood were observed, their magnitude was small.

Subjective adulthood was relatively stable across waves: 87.8% of participants who identified as fully adult at T1 remained in this category at T2, whereas 28.4% of those classified as in-between/not adult at T1 transitioned to the fully adult category at T2 (Supplementary Table S4). The overall distribution did not change significantly across waves, McNemar *χ*^2^(1) = 1.72, *p* = 0.190. Overall, these findings indicate that substantial rank-order stability coexisted with limited mean-level change across the follow-up period.

### 3.5 Longitudinal associations between psychological stress burden and developmental outcomes (H1)

The autoregressive SEMs examined whether baseline stress burden prospectively predicted identity development, resilience-related resources, and adulthood outcomes after controlling for baseline levels of the respective outcomes and the prespecified covariates (Table 2). The autoregressive SEMs examined whether baseline psychological stress burden prospectively predicted identity development, resilience-related resources, and adulthood outcomes after controlling for baseline levels of the respective outcomes and the prespecified covariates (Table 2). Model-fit indices were CFI = 0.919, TLI = 0.861, RMSEA = 0.063, and SRMR = 0.071 for the joint identity model; CFI = 0.849, TLI = 0.799, RMSEA = 0.069, and SRMR = 0.055 for the resilience-related resources model; and CFI = 0.846, TLI = 0.815, RMSEA = 0.065, and SRMR = 0.058 for the objective-adulthood model. The subjective-adulthood probit model showed CFI = 0.984, TLI = 0.993, RMSEA = 0.027, and SRMR = 0.050. Within the joint identity model, higher baseline psychological stress burden predicted greater ruminative exploration (*β* = 0.12, *p* = 0.028, pFDR = 0.047) and lower commitment making (*β* = −0.13, BH-FDR-adjusted *p* = 0.033), and lower identification with commitment (*β* = −0.12, BH-FDR-adjusted *p* = 0.047). Associations with exploration in breadth and exploration in depth were not statistically significant after correction for multiple testing.

**Table 2:** Prospective longitudinal associations of baseline psychological stress burden with developmental outcomes at follow-up (H1).

T2 outcome	*β*	Robust SE	95% CI	*p*	BH–FDR p	*R* ^2^
Exploration in breadth	−0.04	0.05	[−0.15, 0.06]	0.420	0.420	0.21
Commitment making	−0.13	0.05	[−0.23, −0.04]	0.007	0.033	0.35
Exploration in depth	−0.10	0.05	[−0.20, 0.00]	0.060	0.075	0.24
Identification with commitment	−0.12	0.05	[−0.23, −0.02]	0.025	0.047	0.27
Ruminative exploration	0.12	0.05	[0.01, 0.22]	0.028	0.047	0.27
Resilience-related resources	−0.02	0.04	[−0.11, 0.07]	0.639	—	0.56
Objective adulthood	−0.01	0.03	[−0.06, 0.05]	0.824	—	0.77
Subjective adulthood	−0.01	0.07	[−0.15, 0.11]	0.779	—	0.69

*β*, standardized coefficient; SE, standard error; CI, onfidence interval; BH–FDR, Benjamini–Hochberg false discovery rate correction. All models controlled for the corresponding T1 outcome and prespecified sociodemographic covariates. The five identity dimensions were estimated jointly in an autoregressive SEM. Resilience-related resources and objective adulthood were estimated in separate autoregressive SEMs. Subjective adulthood was estimated using a probit SEM with WLSMV estimation; its *R*^2^ refers to the proportion of variance explained on the underlying latent-response scale. BH–FDR correction was applied to the five focal DIDS coefficients. *N* = 831.

Psychological stress burden was not prospectively associated with resilience-related resources, objective adulthood attainment, or subjective adulthood (all *ps* > 0.05). Thus, H1 was partially supported: higher baseline psychological stress burden was prospectively associated with selected identity-development processes, whereas no evidence emerged for prospective associations with resilience-related resources or adulthood attainment.

Inverse-probability-of-retention weighted analyses produced estimates that were highly similar in direction and magnitude to those of the primary models (Supplementary Table S3). The only difference in statistical significance was observed for exploration in depth, for which the negative association with psychological stress burden reached the BH-FDR-adjusted significance threshold in the weighted analysis (*β* = −0.11, BH-FDR-adjusted *p* = 0.049). All other substantive conclusions were unchanged.

Component-specific sensitivity analyses further examined the three indicators of psychological stress burden entered simultaneously as separate predictors (Supplementary Table S6). The identity associations were most consistently observed for perceived stress: PSS-10 prospectively predicted lower commitment making, exploration in depth, and identification with commitment after BH-FDR correction. IADQ symptoms were additionally associated with greater exploration in depth, whereas none of the IADQ-PC associations remained significant after correction. None of the three stress indicators prospectively predicted resilience-related resources, objective adulthood, or subjective adulthood.

### 3.6 Longitudinal associations between resilience-related resources and developmental outcomes (H2)

Autoregressive SEMs were used to examine whether baseline resilience-related resources prospectively predicted developmental and adulthood outcomes after accounting for baseline levels of the respective outcomes, baseline psychological stress burden, and the prespecified covariates (Table 3). Autoregressive SEMs were used to examine whether baseline resilience-related resources prospectively predicted developmental and adulthood outcomes after accounting for baseline levels of the respective outcomes, baseline psychological stress burden, and the prespecified covariates (Table 3). Model-fit indices were CFI = 0.968, TLI = 0.868, RMSEA = 0.061, and SRMR = 0.035 for the joint identity model and CFI = 0.847, TLI = 0.809, RMSEA = 0.065, and SRMR = 0.057 for the objective-adulthood model. The subjective-adulthood probit model showed CFI = 0.984, TLI = 0.993, RMSEA = 0.027, and SRMR = 0.050. Within the joint identity model, higher resilience-related resources predicted greater exploration in breadth (*β* = 0.29), commitment making (*β* = 0.22), exploration in depth (*β* = 0.30), and stronger identification with commitment (*β* = 0.27; all BH-FDR-adjusted *ps* < 0.001), whereas its association with ruminative exploration was not significant (*β* = −0.05, *BH-FDR-adjusted p* = 0.127).

**Table 3:** Prospective longitudinal associations of baseline resilience-related resources with developmental outcomes at follow-up (H2).

T2 outcome	*β*	Robust SE	95% CI	*p*	BH–FDR *p*	*R* ^2^
Exploration in breadth	0.29	0.04	[0.21, 0.37]	<0.001	<0.001	0.31
Commitment making	0.22	0.04	[0.15, 0.29]	<0.001	<0.001	0.44
Exploration in depth	0.30	0.04	[0.23, 0.38]	<0.001	<0.001	0.36
Identification with commitment	0.27	0.04	[0.19, 0.35]	<0.001	<0.001	0.38
Ruminative exploration	−0.05	0.04	[−0.12, 0.02]	0.127	0.127	0.29
Objective adulthood	0.03	0.03	[−0.02, 0.08]	0.250	—	0.77
Subjective adulthood	0.07	0.05	[−0.02, 0.17]	0.106	—	0.70

*β*, standardized coefficient; SE, standard error; CI, confidence interval; BH–FDR, Benjamini–Hochberg false discovery rate correction. All models controlled for the corresponding T1 outcome, baseline psychological stress burden, and prespecified sociodemographic covariates. The five identity dimensions were estimated jointly in an autoregressive SEM. Objective and subjective adulthood were estimated in separate autoregressive SEMs. Subjective adulthood was estimated using a probit SEM with WLSMV estimation; its *R*^2^ refers to the proportion of variance explained on the underlying latent-response scale. BH–FDR correction was applied to the five focal DIDS coefficients. *N* = 831.

No significant prospective associations were found between baseline resilience-related resources and either objective adulthood attainment (*β* = 0.03, *p* = 0.250) or subjective adulthood (*β* = 0.07, *p* = 0.106). Thus, H2 was partially supported, indicating that resilience-related resources prospectively predicted four identity-development processes but did not predict ruminative exploration or either indicator of adulthood attainment.

Inverse-probability-of-retention weighted sensitivity analyses yielded the same substantively pattern (Supplementary Table S3). Resilience-related resources remained positively associated with exploration in breadth, commitment making, exploration in depth, and identification with commitment, whereas associations with ruminative exploration, objective adulthood, and subjective adulthood remained nonsignificant.

## 4 Discussion

The present study examined developmental adaptation among emerging adults over a one-year period marked by continued societal uncertainty associated with multiple concurrent global crises. Overall, the findings revealed substantial rank-order stability in identity processes, resilience-related resources, and adulthood indicators, accompanied by relatively limited mean-level change. Ruminative exploration decreased slightly over time, whereas small increases were observed in several domains of objective adulthood. Importantly, these two forms of longitudinal continuity and change are conceptually distinct: relatively large autoregressive coefficients indicate that participants tended to maintain their relative positions on the study variables over time, but do not imply an absence of group-level developmental change.

Against this background, baseline psychological stress burden showed selective prospective associations with identity development, predicting greater ruminative exploration and lower commitment making and identification with commitment at follow-up, while showing no prospective association with resilience-related resources or either indicator of adulthood after accounting for baseline levels and sociodemographic covariates. Higher baseline resilience-related resources, in contrast, was prospectively associated with more adaptive identity development. Taken together, the findings indicate selective prospective associations of psychological stress burden and resilience-related resources with identity development rather than broader adulthood attainment.

The significant autoregressive coefficients observed across developmental outcomes indicate moderate-to-high rank-order stability over the one-year follow-up. This pattern is consistent with longitudinal research showing considerable continuity in individual differences in identity processes during emerging adulthood (46, 47). At the same time, mean-level analyses indicated little change in most identity dimensions and resilience-related resources, alongside a small decrease in ruminative exploration and modest increases in several domains of objective adulthood. Thus, stability in participants’ relative standing coexisted with limited but detectable group-level developmental change. Because the longitudinal models accounted for baseline levels of the respective outcomes, the prospective associations identified in the present study reflect prediction of follow-up individual differences beyond their prior levels.

From a developmental systems perspective, such stability is theoretically meaningful because development reflects the cumulative product of dynamic interactions between individuals and their environments rather than simply reflecting immediate responses to changing contextual conditions (48). Identity formation, in particular, is understood as an ongoing developmental process that unfolds through repeated cycles of exploration and commitment over many years rather than through abrupt shifts in response to isolated life events (9, 10). Consequently, although societal crises may temporarily increase psychological distress, their prospective associations with broader developmental adaptation may be more limited than would be expected based on cross-sectional associations. These findings are also consistent with resilience research demonstrating that adaptive development often persists despite substantial adversity. Masten (49) argued that resilience frequently reflects the operation of normative developmental systems—an idea captured in the concept of *ordinary magic*—whereby ordinary psychological, familial, and social resources enable continued adaptation under challenging circumstances. Moreover, the observed temporal stability aligns with recent longitudinal studies reporting moderate-to-high continuity in identity dimensions during emerging adulthood despite substantial life transitions and contextual challenges (12, 47, 50). Given that identity development and adulthood attainment unfold over multiple years, the one-year observation window may additionally have limited the magnitude of detectable developmental change.

### 4.1 Psychological stress burden and identity development

Psychological stress burden showed prospective associations with selected identity processes, providing partial support for H1. Higher baseline stress burden predicted greater ruminative exploration and lower commitment making and identification with commitment, whereas associations with exploration in breadth and exploration in depth did not remain statistically significant after correction for multiple testing. Stress burden was also unrelated to subsequent resilience-related resources, objective adulthood, and subjective adulthood.

This pattern suggests that identity processes may differ in their sensitivity to contextual stressors. Identity exploration represents an active, cognitively demanding, and emotionally intensive process requiring individuals to evaluate alternatives, integrate new experiences, and make personally meaningful commitments (9, 10). Sustained stress may interfere with these processes by placing demands on the cognitive and emotional resources involved in adaptive identity work. Consistent with this possibility, higher psychological stress burden was prospectively associated with greater ruminative exploration—a repetitive and less adaptive form of identity exploration—and with weaker commitment-related processes. However, because the present study did not directly assess the mechanisms linking stress burden to identity development, such explanations remain theoretical rather than demonstrated.

Component-specific sensitivity analyses provided additional nuance. When perceived stress, adjustment-disorder symptoms, and polycrisis-related adjustment difficulties were entered simultaneously, the prospective identity associations were most consistently evident for perceived stress. Higher perceived stress independently predicted lower commitment making, exploration in depth, and identification with commitment after correction for multiple testing. Adjustment-disorder symptoms were additionally associated with greater exploration in depth, whereas no association involving polycrisis-related adjustment difficulties remained significant after correction (Supplementary Table S6). Thus, the component-specific findings do not indicate that all three indicators contributed equally to the prospective identity associations captured by the latent psychological stress-burden factor. At the same time, they should not be interpreted as demonstrating that perceived stress is uniquely or causally responsible for subsequent identity development. The indicators capture related but distinct aspects of psychological stress burden, and their differential associations may reflect differences in their conceptual content, variability, or proximity to the developmental processes examined.

Several mechanisms could potentially account for these associations. Sustained stress may place demands on the cognitive and emotional resources required to evaluate alternatives and maintain commitments, while heightened uncertainty may promote repetitive self-focused processing characteristic of ruminative exploration. Experimental evidence in young adults indicates that psychosocial and cognitive stress can impair cognitive flexibility and related executive functions, including working memory and verbal fluency (51), providing one potential pathway through which stress may be associated with identity exploration and commitment formation. Adaptive functioning under stress may also depend on the flexible interplay between relatively automatic and controlled processes (52). However, cognitive flexibility, executive functioning, and these regulatory processes were not assessed in the present study and should therefore be regarded as potential explanatory mechanisms rather than demonstrated mediators.

Recent longitudinal findings likewise suggest that the association between stressful experiences and identity development is more nuanced than originally proposed by identity theory. Whereas de Moor et al. (53) found no evidence that selected stressful life events predicted regressive identity change in adolescence, Wong and Hamza (54) demonstrated that increases in specific stressors were associated with greater ruminative exploration and lower commitment making in emerging adulthood. The present findings add to this literature by showing that an individual-level psychological stress burden measured within a broader polycrisis context prospectively predicted selected identity processes after accounting for their baseline levels.

In contrast, neither objective nor subjective adulthood indicators was prospectively associated with psychological stress burden. Objective adulthood primarily reflects enduring social roles and responsibilities, such as educational completion, employment, financial independence, and long-term relationships, which typically evolve over several years rather than within a single year. Likewise, subjective adulthood reflects a relatively stable self-perception that develops gradually through accumulated developmental experiences (1, 43). Consequently, psychological stress burden may be more closely associated with ongoing identity negotiations than with broader indicators of adulthood attainment over a relatively short observation period.

Taken together, the findings suggest that psychological stress experienced within a polycrisis context may be more closely associated with how emerging adults negotiate and consolidate their identities than with whether they attain broader markers of adulthood over a relatively short period. This distinction between the relative malleability of identity processes and the greater temporal stability of adulthood attainment has received limited attention within the emerging literature on polycrisis and developmental adaptation and may help explain why contemporary societal uncertainty may be associated with *how* emerging adults negotiate their identities more readily than *whether* they progress toward adulthood itself.

### 4.2 Resilience-related resources and identity development

The present findings provided partial support for H2. Importantly, the KOP-26 operationalizes resilience-related personal, family, and social resources rather than resilience as a dynamic process of successful adaptation under adversity. Accordingly, the findings concern the prospective role of resilience-related resources and should not be interpreted as direct evidence that resilience as a process produced developmental adaptation.

After accounting for baseline identity levels, psychological stress burden, and sociodemographic covariates, higher resilience-related resources prospectively predicted greater exploration in breadth, commitment making, exploration in depth, and identification with commitment. The association with ruminative exploration was not statistically significant. This pattern is noteworthy because resilience-related resources were associated not only with commitment processes but also with the two adaptive forms of exploration. Rather than suggesting that these resources primarily facilitate commitment consolidation, the findings point to a broader association with adaptive identity work, encompassing both active exploration of alternatives and stronger commitment formation and identification.

One possible interpretation is that the personal, family, and social resources captured by the KOP-26 provide a supportive basis for engaging with the developmental tasks of emerging adulthood. Identity development requires individuals both to explore alternatives and to establish and maintain meaningful commitments. Resources related to self-regulation social support, perceived competence, and adaptive coping may facilitate engagement in these processes even under conditions of uncertainty (24, 30). Adaptive functioning may furthermore depend not simply on stronger controlled regulation, but on the flexible coordination of automatic and controlled processes (52). Contrary to H2, resilience-related resources did not prospectively predict either objective or subjective adulthood after baseline adulthood, psychological stress burden, and covariates were taken into account. This distinction is important. Although resilience-related resources were consistently associated with identity processes, their prospective associations did not extend to broader markers of adulthood attainment within the one-year interval. One possible explanation is that objective adulthood depends substantially on structural and contextual opportunities—including education, employment, financial independence, and relationship transitions—that unfold within particular social and institutional contexts and often develop over extended periods (1, 43). Similarly, subjective adulthood reflects an evolving self-perception grounded in accumulated experiences of independence, responsibility, and social-role transitions rather than a single developmental achievement (1, 43). The absence of prospective associations with these outcomes over 1 year may therefore reflect their relatively cumulative nature rather than an absence of relevance of resilience-related resources to the broader transition to adulthood.

More broadly, the present pattern is consistent with developmental conceptualizations of resilience as arising from adaptive processes and resources operating across multiple levels of the individual’s ecology, rather than constituting a fixed individual characteristic (29, 49, 55). From this perspective, the personal, family, and social resources captured by the KOP-26 may be relevant to particular developmental processes without necessarily predicting change across all domains of functioning. In the present study, their prospective associations were concentrated within identity development, encompassing both adaptive exploration and commitment processes. Whereas many studies have demonstrated that resilience predicts lower emotional distress and greater life satisfaction (33, 56), considerably fewer have examined developmental milestones such as adulthood attainment. Our findings suggest that the developmental correlates of resilience-related resources may be domain-specific: over the one-year interval examined here, they were evident for identity processes but not for broader markers of adulthood attainment. This distinction underscores the importance of examining specific developmental domains rather than treating developmental adaptation as a unitary outcome.

### 4.3 Theoretical implications

The present findings contribute to several areas of developmental research by suggesting that psychological stress burden and resilience-related resources were prospectively associated with specific aspects of identity development against a background of considerable temporal stability. Much of the recent literature on the psychological consequences of the COVID-19 pandemic and other global crises has understandably emphasized increased distress, uncertainty, and vulnerability among young people (57, 58). Although these findings are well supported, elevated distress does not necessarily imply broad changes across all domains of developmental functioning. In the present study, rank-order stability was evident across developmental outcomes, mean-level changes were generally small, and the focal prospective associations were concentrated primarily within identity development.

These findings also refine developmental theories of identity formation by highlighting differences in the temporal sensitivity of specific developmental domains. Psychological stress burden was associated with greater ruminative exploration and weaker commitment-related processes, whereas resilience-related resources were associated with exploration in breadth, exploration in depth, commitment making, and identification with commitment. This pattern supports process-oriented models of identity development, which conceptualize exploration and commitment as distinct but interacting developmental mechanisms rather than opposite ends of a single continuum (9, 10). In particular, the contrast between adaptive forms of exploration and ruminative exploration underscores the importance of distinguishing among qualitatively different ways of engaging with identity uncertainty.

The findings also help locate the study within a macro-to-micro account of polycrisis. Societal crises operate at a macrosystemic level by reshaping opportunity structures and sources of uncertainty, whereas their developmental implications depend on individual exposure, appraisal, adjustment responses, and available resources. The psychological stress-burden construct used here therefore captures an individual-level psychological correlate of living within a broader polycrisis context, rather than objective exposure to polycrisis itself. Accordingly, the findings should not be interpreted as estimating a direct effect of polycrisis on developmental outcomes.

More broadly, the present findings are compatible with developmental resilience perspectives emphasizing that adaptation under adversity depends on resources embedded within individuals and their social environments (29, 30). Contemporary emerging adults often encounter prolonged exposure to multiple interacting stressors, including public health crises, economic instability, geopolitical conflict, and climate-related uncertainty. Our findings suggest that resilience-related resources remain relevant under these conditions of chronic societal uncertainty, supporting adaptive developmental functioning even when stressors are cumulative rather than isolated. Rather than viewing polycrisis solely as a threat to development, developmental psychology may benefit from adopting a more balanced perspective that recognizes both vulnerability and the remarkable capacity of normative developmental systems to sustain adaptation over time (59).

### 4.4 Practical implications

The present findings have several practical implications for professionals working with emerging adults. First, the results suggest that interventions should extend beyond efforts to reduce psychological distress and should also promote developmental resources that facilitate successful identity formation and the transition to adulthood. Psychological stress burden was prospectively associated with selected identity processes, whereas resilience-related resources showed consistent prospective associations with adaptive exploration and commitment processes. These findings identify resilience-related resources and identity processes as potentially relevant targets for developmental support, although intervention studies are required before concluding that modifying these resources would produce improvements in identity development (30).

Universities represent particularly important settings for such interventions because many emerging adults experience major educational, occupational, and interpersonal transitions during higher education. Programmes that promote self-regulation, adaptive coping, emotion regulation, goal setting, and reflective identity exploration may therefore complement conventional symptom-focused services. Likewise, counselling and mental health services may benefit from incorporating identity-oriented approaches alongside traditional symptom-focused interventions. Facilitating commitment formation, supporting career and relationship decision-making, and helping young people integrate stressful experiences into coherent life narratives may enhance both psychological wellbeing and developmental adaptation (60).

At a broader level, the findings support preventive approaches that strengthen protective resources before psychological difficulties become clinically significant. Emerging adulthood represents a sensitive developmental period during which relatively small improvements in resilience and self-regulatory functioning may have long-term consequences for identity development, educational attainment, occupational adjustment, and later mental health (59). Consequently, resilience-related resources may represent promising targets for programmes designed to support developmental functioning during emerging adulthood.

### 4.5 Strengths and limitations

Several strengths enhance confidence in the present findings. The study employed a large nationwide longitudinal sample, enabling prospective examination of developmental adaptation during a period of prolonged societal uncertainty. Unlike most previous research, which has focused on individual crises or solely on psychological distress, the present study simultaneously assessed individual life stressors, polycrisis-related adjustment difficulties, identity development, resilience, and both subjective and objective indicators of adulthood. The autoregressive modelling approach accounted for baseline levels of the respective outcomes and a broad set of sociodemographic covariates, allowing the analyses to examine whether baseline psychological stress burden and resilience-related resources predicted follow-up individual differences beyond prior levels. The distinction between rank-order stability and mean-level change further provided complementary information about developmental continuity across the one-year interval.

Nevertheless, several limitations should be acknowledged. First, the study included only two measurement occasions separated by approximately 1 year. Although autoregressive models permit prospective associations with follow-up outcomes to be estimated while accounting for baseline levels, two-wave data cannot characterize developmental trajectories, estimate individual growth parameters, or establish the shape of developmental change. The autoregressive coefficients should therefore be interpreted as indicators of rank-order stability rather than evidence that developmental change did not occur. Moreover, the one-year interval may have been insufficient to detect changes in relatively stable outcomes such as adulthood attainment. Studies incorporating three or more measurement occasions over longer periods are needed to examine developmental change more directly. Second, all measures relied on self-report, raising the possibility of shared-method variance and response biases. The dichotomization of subjective adulthood represents an additional measurement limitation. Participants who described themselves as “in-between” were combined with those who did not consider themselves adults because very few participants endorsed the latter category at follow-up. Although this was necessary for stable statistical estimation, it obscures a theoretically meaningful distinction, as feeling “in-between” is itself a defining feature of emerging adulthood (1). Future studies with sufficient observations across all response categories should retain this distinction. Third, the study was nationwide but should not be interpreted as fully representative of all emerging adults in Poland. Moreover, developmental experiences may differ across sociocultural contexts characterised by different educational systems, labour markets, welfare policies, and exposure to societal crises. Replication in other national contexts is therefore needed. Fourth, despite the longitudinal design, the observational nature of the study precludes firm causal conclusions. The models establish temporal ordering between baseline predictors and follow-up outcomes while controlling for baseline outcome levels, but unmeasured time-varying or stable confounding cannot be excluded. Finally, attrition was selective with respect to several baseline characteristics, although standardized differences in the principal psychological variables were generally small. Inverse-probability-of-retention weighted sensitivity analyses yielded estimates highly similar in direction and magnitude to those of the primary models. The only change in statistical significance concerned exploration in depth in H1, which reached the BH-FDR-adjusted significance threshold in the weighted analysis but not in the primary model. Thus, the principal conclusions were robust to the IPW analysis, although differential attrition may still limit the generalizability of the longitudinal findings.

### 4.6 Future research

The present findings raise several important questions for future research. First, studies spanning longer developmental periods are needed to determine whether psychological stress burden and resilience-related resources are associated with rates or patterns of developmental change rather than only with subsequent outcomes after baseline adjustment. Latent growth models and other longitudinal approaches may also identify heterogeneity in patterns of adaptation over time (61).

Second, cross-cultural longitudinal studies would help establish whether the observed pattern generalises across countries that differ in economic conditions, educational systems, and exposure to societal crises. Such comparisons may clarify the extent to which developmental resilience reflects universal developmental processes or context-specific resources (28).

Third, future research should investigate the mechanisms through which resilience-related resources are prospectively associated with adaptive identity development. Potential mediators include emotion regulation, future orientation, self-efficacy, optimism, and executive functioning (24). Finally, greater attention should be devoted to interpersonal protective factors, including family relationships, peer support, romantic partnerships, and mentoring relationships, which may strengthen resilience and promote adaptive developmental trajectories during periods of prolonged societal uncertainty. Future studies should also examine whether such resources become associated with adulthood attainment over longer developmental intervals than the one-year period examined here.

## 5 Conclusion

Emerging adulthood is characterized by ongoing identity development and changing social roles, and contemporary cohorts are navigating these developmental tasks amid prolonged societal uncertainty. Across the one-year follow-up, the developmental outcomes examined showed significant rank-order stability, while mean-level changes were generally small; ruminative exploration decreased slightly and several domains of objective adulthood showed modest increases. Higher baseline psychological stress burden prospectively predicted greater ruminative exploration and lower commitment making and identification with commitment, but was not associated with subsequent resilience-related resources or adulthood attainment. Higher baseline resilience-related resources prospectively predicted greater exploration in breadth and depth, commitment making, and identification with commitment, but did not predict ruminative exploration or either objective or subjective adulthood. Thus, the prospective associations observed in the present study were concentrated primarily within identity development rather than broader adulthood attainment. Because two-wave observational data cannot establish developmental trajectories, mechanisms, or causal effects, longer multiwave studies are needed to determine how psychological stress burden and resilience-related resources relate to developmental adaptation across the transition to adulthood.

## Data Availability

The raw data supporting the conclusions of this article will be made available by the authors, without undue reservation.
